# O5-1 Physical activity promotion in long term illness patients: Preliminary results on a French national program

**DOI:** 10.1093/eurpub/ckac094.033

**Published:** 2022-08-29

**Authors:** Nicolas Salesse A Robin, Jonathan Maury, Chloé Zaffuto, Gauthier Ruspini, Amélie Fuchs, Lucile Bigot, Aline Herbinet

**Affiliations:** Research, Development and innovation department, SAS MOOVEN, Montpellier, France

**Keywords:** Long-term illness, practice standardisation, digital tools, innovative care pathway, evaluation

## Abstract

**Issue/problem:**

Since 2016, French doctors are allowed to prescribe adapted physical activity (APA) to patients with long-term illness (ALD) through the ‘sport sur ordonnance' program. Despite the goal of promoting physical activity in ALD patients, health authority recent reports unanimously highlight organisational and funding difficulties. Whereas most of the funding effort is based on national or mutual insurance companies, our intervention proposes to fix the organisational difficulties. We provided an optimized care pathway coordinated by an APA professional and an innovative online platform to ease medical prescription, patients access and follow-up to APA.

**Problem description:**

Three main problems have been identified: Diagnostic heterogeneity, APA accessibility and Program evaluation. To fix these issues, the proposed intervention firstly included an initial evaluation based on a standardized diagnostic. Secondly, we evaluated APA structures following functional specifications and referenced those succeeding the criteria to ensure an optimized patients' orientation toward an adapted care service. Finally, pre- and post-care bio-psycho-social tests were mandatory. Interviews and evaluation data were stored via questionnaires on our securized platform for further analyses.

**Results:**

2200 patients benefited from our program, and 116 yet finished the whole 2-years program. Patients description: 64 yo, 66% women, 35% cancer, 14% diabetes, 11% heart failure and 9% mental illnesses. 96% declared being motivated to maintain their physical activity after the program and reported a 4.8/5 in rating their APA care. Significant pre-post enhancements suggest that our program succeed in promoting and facilitating regular APA practice in ALD patients.

**Lessons:**

The ecosystem built around insurance companies (APA professionals, doctors and patients) provides an appropriate response to the health policy dedicating to promote a regular APA practice for ALD patients. However, the expected efficiency depends on a structured, organized and innovative system. Firstly, evaluating the medicoeconomic impact is necessary to ensure the sustainability of this national policy. Systematically measuring the pre- and post-care intervention should allow reaching this objective. Secondly, we learned that the necessary next steps will rely on time saving and quality increase. We propose automatized detailed and patient specific APA prescription following HAS guidelines and automatized orientation toward the most adapted APA professional.

